# Endogenous Retroviruses and Human Evolution

**DOI:** 10.1002/cfg.216

**Published:** 2002-12

**Authors:** Konstantin Khodosevich, Yuri Lebedev, Eugene Sverdlov

**Affiliations:** Shemyakin–Ovchinnikov Institute of Bioorganic Chemistry Russian Academy of Science 16/10 Miklukho-Maklaya Moscow 117997 Russia

## Abstract

Humans share about 99% of their genomic DNA with chimpanzees and bonobos; thus, the differences between these species are unlikely to be in gene content but could be caused by inherited changes in regulatory systems. Endogenous retroviruses (ERVs) comprise ∼ 5% of the human genome. The LTRs of ERVs contain many regulatory sequences, such as promoters, enhancers, polyadenylation signals and factor-binding sites. Thus, they can influence the expression of nearby human genes. All known human-specific LTRs belong to the HERV-K (human ERV) family, the most active family in the human genome. It is likely that some of these ERVs could have integrated into regulatory regions of the human genome, and therefore could have had an impact on the expression of adjacent genes, which have consequently contributed to human evolution. This review discusses possible functional consequences of ERV integration in active coding regions.


**Comparative and Functional Genomics**
*Comp Funct Genom* 2002; **3**: 494–498.
Published online in Wiley InterScience (www.interscience.wiley.com). **DOI:** 10.1002/cfg.216

**Conference Review**

### Endogenous retroviruses and human evolution

Konstantin Khodosevich*, Yuri Lebedev and Eugene Sverdlov
*Shemyakin-Ovchinnikov Institute of Bioorganic Chemistry, Russian Academy of Science, 16/10 Miklukho-Maklaya, 117997 Moscow, Russia*

**Correspondence to: Konstantin Khodosevich, Shemyakin-Ovchinnikov Institute of Bioorganic Chemistry, Russian Academy of Science, 16/10 Miklukho-Maklaya, 117997 Moscow, Russia. E-mail: kosthob@humgen.siobc.ras.ru*

Received: 4 September 2002
Accepted: 9 October 2002

### Abstract

**Humans share about 99% of their genomic DNA with chimpanzees and bonobos; thus, the differences between these species are unlikely to be in gene content but could be caused by inherited changes in regulatory systems. Endogenous retroviruses (ERVs) comprise ∼ 5% of the human genome. The LTRs of ERVs contain many regulatory sequences, such as promoters, enhancers, polyadenylation signals and factor-binding sites. Thus, they can influence the expression of nearby human genes. All known human-specific LTRs belong to the HERV-K (human ERV) family, the most active family in the human genome. It is likely that some of these ERVs could have integrated into regulatory regions of the human genome, and therefore could have had an impact on the expression of adjacent genes, which have consequently contributed to human evolution. This review discusses possible functional consequences of ERV integration in active coding regions. Copyright © 2002 John Wiley & Sons, Ltd.**

**Keywords: human evolution; human endogenous retroviruses; long terminal repeats; human genome; transposition; gene expression; regulatory sequences**

### Introduction

Humans tend to think of themselves as being something special, truly different from other animals, but at the molecular level it is obvious that we are very similar to the chimpanzee species. The average DNA sequence difference between human and chimpanzee is only 1.24% [7] and probably only 0.5% in active coding regions [9]. In the light of this data, one could ask the question: what genetic differences separated us from apes and made us human? So far, only a few significant human-specific genomic features have been identified (reviewed in [8]). Eighteen of the 23 pairs of modern human chromosomes are virtually identical to chimpanzee chromosomes, with the most significant differences between the human and chimpanzee karyotypes being a telomeric fusion of chimpanzee chromosomes 12 and 13 to form human chromosome 2, and pericentric inversions in several chromosomes [30]. Among known human-specific differences in gene coding regions, the most important is the inactivation of the human CMP-*N*-acetylneuraminic acid (CMP-Neu5Ac) hydroxylase gene, which leads to an absence of CMP-*N*-glycolylneuraminic acid (CMP-Neu5Gc) on the surface of all human cells and, consequently, an increased quantity of CMP-Neu5Ac [10]. However, as early as 1975, King and Wilson concluded [15]: 'their (human and chimpanzee) macromolecules are so alike that regulatory mutations may account for their biological differences' and it is most probable that the differences between human and chimpanzee could be caused by differences in the regulatory systems of their genomes. To date, there are only a few examples of differences in regulatory regions between these species [8,12]. Among the best candidates that could play a role in generating such differences in regulatory regions are transposable elements and, of these, especially retroelements (REs).

Copyright © 2002 John Wiley & Sons, Ltd.

### Retroelements — Endogenous retroviruses

Retroelements are mobile elements that transpose via an RNA intermediate. There are three main groups of REs: long interspersed elements (LINEs), short interspersed elements (SINEs) and long terminal repeat (LTR) elements. Endogenous retroviruses (ERVs), which are members of the LTR elements, are the most complex REs. They are widespread throughout vertebrates and the number of ERVs in a haploid genome could be between tens of copies and several tens of thousands of copies [11]. A full-length provirus consists of three major genes, *gag*, *pol* and *env*, and is flanked by LTRs (Figure 1A). LTRs contain many regulatory sequences (Figure 1B), such as promoter, enhancer, polyadenylation signal and factor-binding sites.

REs could influence gene regulation by expressing their retroviral genes, inducing genomic rearrangements, providing new regulatory sequences or simply by disrupting gene functions. The involvement of REs in the regulation of gene expression has been demonstrated in a number of studies [4,18,24,25].

It is universally recognized that ERVs are the remnants of exogenous retroviral germ cell infections [4,18,24,25]. After invasion of the host cell, viral RNA is converted into cDNA, which then integrates into the host genome. Over time, many ERVs have disappeared from host genomes due to homologous recombination between two LTRs (generating solitary LTRs), as demonstrated by the lower number of full-length proviruses than solitary LTRs in modern vertebrate genomes.

### Human ERVs

Over 41% of the human genome is represented by retroelements; 13% LINEs, 20% SINEs and 8%

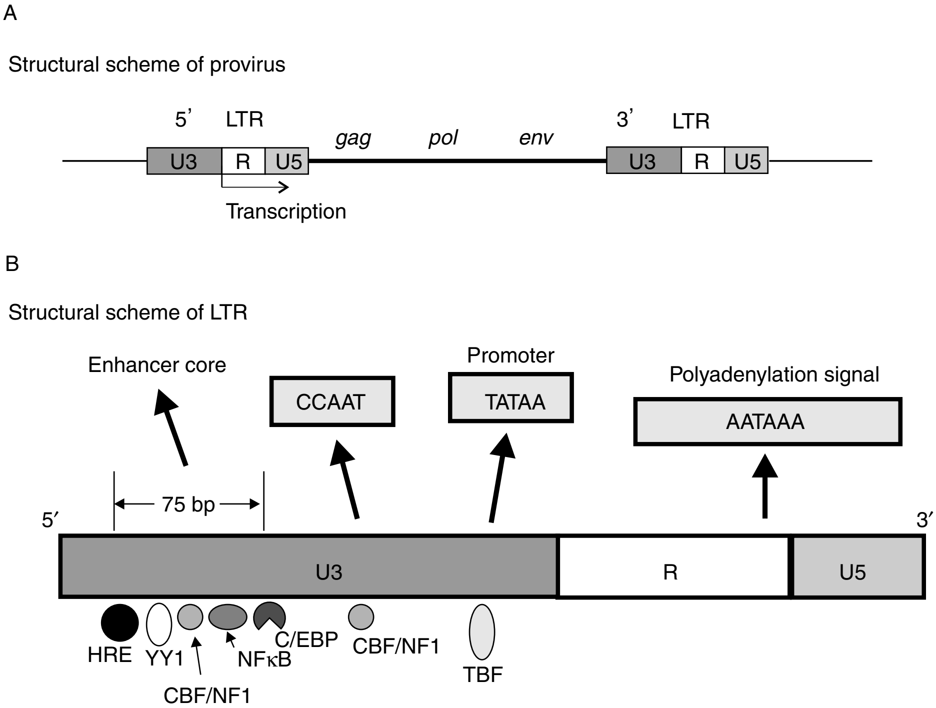

**Figure 1.** The structure of HERV-K as an example of ERVs. (A) Structural scheme of provirus. LTR (long terminal repeat) consisting of stretches designated U3, R and U5; *gag*, group-specific antigen gene; *pol*, polymerase gene; *env*, envelope gene. The arrow indicates a transcription start point. (B) Structural scheme of LTR with the positions of the promoter, polyadenylation signal, enhancer core and putative factor-binding sites marked. TBF, TATA-box binding factor; CBF/NF1, CCAAT binding factor/nuclear factor 1; HRE, hormone-response element; C/EBP, core/enhancer binding protein; NF$\kappa$B, nuclear factor $\kappa$B; YY1, yin yang-1

Copyright © 2002 John Wiley & Sons, Ltd. 

LTR elements [16]. Most of the LTR elements are from ERVs. Human ERVs (HERVs) are specific for primate genomes. Their expression has been found in almost every human tissue and organ, including placenta and embryonic tissues, different tumours, lung and kidney [18,23,25]. HERV transcripts commonly contain many mutations in their ORFs and do not code for any functional proteins. However, some proviruses have intact ORFs, which are evidenced by the presence of retroviral proteins or the detection of their enzymatic activities in some human tissues [2,3,18,26]. Furthermore, in addition to retroviral proteins, virus-like particles (VLPs) have been found in human tissues, which were shown to be able to bud from the cell membrane, but to be unable to infect cells [18]. Recently, Mi *et al.* [22] showed that the HERV-W Env protein (also called syncytin) expresses in placenta and takes part in the syncytiotrophoblast formation. Since syncytin has been detected only in the primate lineage and not in other mammals, it could explain differences in placental biology between primates and other mammals. It is not known whether HERVs retained a transpositional capability after the divergence of the human and chimpanzee evolutionary branches, but the presence of human-specific HERVs suggests that some of them were still active.

All known human-specific HERVs belong to HML-2 subfamily of the HERV-K family. This is one of the largest ERV groups in the human genome, represented by about 170 full-length proviruses [27] and 2000 solo LTRs [20]. It has been suggested that HERV-K (HML-2) is the most biologically active retroviral group in the human genome and contains many young members. Some of them are human-specific and, because of their regulatory potential, they could have influenced the expression of adjacent genes and thus contributed to human evolution [2,17,20,28].

Recently, in our laboratory two different methods for a whole-genome comparison of integrations of interspersed repeats between closely related genomes were invented and applied to the genome-wide identification of human-specific HERV-K LTRs [5,19]. Using one of these methods (called targeted genomic differences analysis; TGDA) we found 23 new human-specific LTR members and estimated a lower limit of the total number of them as 67 [5]. Applying another method, known as DiffIR (differences in integration sites of low and medium copy number interspersed repeats), led to the discovery of 11 new human-specific LTR members [19]. On the basis of known human-specific LTR sequences, we created a consensus sequence of the evolutionarily young HERV-K (HML-2) LTRs and searched for similar sequences in the human genome databases. We found $\sim$ 140 LTRs with 97–100% identity to the consensus and checked 19 selected LTRs for their presence in human and non-human primate genomes. Seventeen of these 19 LTRs were human-specific. Since only $\sim$ 90% of the human genome sequence was available at that time, we concluded that the total number of human-specific HERV-K (HML-2) LTRs could be about 140 [6]. We have also shown that there were at least three active groups of HERVs after the divergence of the human and chimpanzee evolutionary branches. These are HERV-K (HML-2), with LTRs of groups II-T, HS-a and HS-b, and they are represented by 1, 89 and 53 copies in the human genome, respectively [6].

### The effects of HERVs on gene regulation and their potential contribution to human evolution

Many of the identified human-specific LTRs are located in promoter or enhancer regions, as well as in introns of known or candidate genes [6], e.g. one such LTR is situated in the second intron of the *cbf2* gene (CCAAT-binding factor). Cbf takes part in regulating the expression of many genes, which are involved in various cell processes, such as heat-shock activated genes. Another human-specific LTR is located $\sim$ 6 kb upstream of the transcription start of the *fntb* gene ($\beta$ subunit of CAAX box-farnesyltransferase). Fntb is required for protein farnesylation, which facilitates protein–membrane association and also promotes protein–protein interactions. There are at least 30 known genes that are co-localized with human-specific LTRs, and several other candidate genes; examples are: *ppm1G* (protein phosphatase 1G); *mmp24* (matrix metalloproteinase 24); and *il23a* (interleukin 23, $\alpha$ subunit) [6]. Changes in the expression of such proteins would become apparent, even at the organism level. The identified human-specific LTRs could have influenced the expression of these genes during the process of human and chimpanzee divergence and contributed

Copyright © 2002 John Wiley & Sons, Ltd. 

to human evolution. To date, human-specific LTRs have been detected in the genomes and transcriptomes of various human cancer cells [14,29].

Analyses of individual non-human-specific HERV members have shown their ability to affect the regulation of human genes. Jurka and Kapitonov [13] analysed the leptin receptor (OBR), which is involved in energy expenditure, production of sex hormones and other important biological processes, and found two alternative forms (short and long) of expressed protein. The short form is generated as a result of alternative splicing within a HERV-K (HML-2) LTR and, moreover, this LTR encodes 67 terminal leptin receptor amino acids. Medstrand and Mager analysed the effect of two HERV-E LTRs, one of which had integrated into the 5′ flanking region of the *apoC1* (apolipoprotein C-I) gene and the other into the 5′ flanking region of the *ednrb* gene (endothelin B receptor), on the expression of the adjacent genes [21]. They showed that *apoC1* and *ednrb* have alternative promoters within the LTRs, and that transcription from the alternative *endrb* promoter is much stronger than from the native promoter. Transcription from the *apoC1* LTR promoter is equal to that from native promoter, but the presence of the LTR increases the *apoC1* promoter activity in human and baboons (in this case the LTR plays the role of an enhancer). Recently, we have demonstrated promoter and enhancer activity of human-specific LTRs in reporter gene assays [1]. However, further analysis is needed to verify the hypothesis that ERVs have played a role in human evolution.

To determine the significance of ERVs in human evolution we need to analyse not only young ERV members, but also old ones. Some old members, which existed in all primates and retained transpositional activity after the human–chimpanzee divergence, could have transposed to another locus in the human genome, but not in the chimpanzee genome. Even if such an ERV did transpose in the chimpanzee genome, it is almost impossible that it would transpose to the same site as in the human genome. Furthermore, as a result of mutations, inactive (or silent) proviruses could become transcriptionally (or even transpositionally) active. Thus, old ERVs could express their proteins, form VLP or transpose. Old solitary LTRs could acquire a new functional capacity due to specific mutations in the human genome, e.g. they could become new promoters or enhancers, or alternative splice sites, or factor-binding sites could appear within their sequence. Recently, Schon *et al.* found a subgroup of HERV-W LTRs, which have mutations between the CCAAT-box and TATA-box forming the consensus sequence of the Sp1 binding site [23].

### Conclusion

Retroviral LTRs contain various regulatory sequences in a compact state. The appearance of such elements in a host genome could dramatically change the expression of adjacent genes or even inactivate them. In some cases, the effects of these integrated elements could be of benefit to the host cell. In this way, HERVs could have been an impulsive force in the divergence of human and chimpanzee.

### Acknowledgements

We thank Ilya Yampolski and Nishith Gupta for invaluable comments on the manuscript. The work in the author's laboratory was supported by INTAS-2001-0759 and RFBR-02-04-48614.

### References

1. Akopov SB, Nikolaev LG, Khil PP, Lebedev YB, Sverdlov ED. 1998. Long terminal repeats of human endogenous retrovirus K family (HERV-K) specifically bind host cell nuclear proteins. *FEBS Lett* **421**: 229–233.
2. Barbulescu M, Turner G, Seaman MI, *et al.* 1999. Many human endogenous retrovirus K (HERV-K) proviruses are unique to humans. *Curr Biol* **9**: 861–868.
3. Berkhout B, Jebbink M, Zsiros J. 1999. Identification of an active reverse transcriptase enzyme encoded by a human endogenous HERV-K retrovirus. *J Virol* **73**: 2365–2375.
4. Boeke JD, Stoye JP. 1997. Retrotransposons, endogenous retroviruses, and the evolution of retroelements. In *Retroviruses*, Coffin JM, Hughes SH, Varmus HE (eds). Cold Spring Harbor Laboratory Press: New York; 343–435.
5. Buzdin A, Khodosevich K, Mamedov I, *et al.* 2002. A technique for genome-wide identification of differences in the interspersed repeats integrations between closely related genomes and its application to detection of human-specific integrations of HERV-K LTRs. *Genomics* **79**: 413–422.
6. Buzdin A, Ustyugova S, Khodosevich K, *et al.* 2002. (accepted).
7. Ebersberger I, Metzler D, Schwarz C, Paabo S. 2002. Genomewide comparison of DNA sequences between humans and chimpanzees. *Am J Hum Genet* **70**: 1490–1497.
8. Gagneux P, Varki A. 2001. Genetic differences between humans and great apes. *Mol Phylogenet Evol* **18**: 2–13.
9. Goodman M. 1999. The genomic record of humankind's evolutionary roots. *Am J Hum Genet* **64**: 31–39.

Copyright © 2002 John Wiley & Sons, Ltd. 

10. Hayakawa T, Satta Y, Gagneux P, Varki A, Takahata N. 2001. Alu-mediated inactivation of the human CMP-N-acetylneuraminic acid hydroxylase gene. *Proc Natl Acad Sci USA* **98**: 11 399–11 404.
11. Herniou E, Martin J, Miller K, *et al.* 1998. Retroviral diversity and distribution in vertebrates. *J Virol* **72**: 5955–5966.
12. Huby T, Dachet C, Lawn RM, *et al.* 2001. Functional analysis of the chimpanzee and human apo(a) promoter sequences: identification of sequence variations responsible for elevated transcriptional activity in chimpanzee. *J Biol Chem* **276**: 22 209–22 214.
13. Kapitonov VV, Jurka J. 1999. The long terminal repeat of an endogenous retrovirus induces alternative splicing and encodes an additional carboxy-terminal sequence in the human leptin receptor. *J Mol Evol* **48**: 248–251.
14. Kim HS, Yi JM, Jeon SH. 2001. Isolation and phylogenetic analysis of HERV-K long terminal repeat cDNA in cancer cells. *AIDS Res Hum Retroviruses* **17**: 987–990.
15. King MC, Wilson AC. 1975. Evolution at two levels in humans and chimpanzees. *Science* **188**: 107–116.
16. Lander ES, Linton LM, Birren B, *et al.* 2001. Initial sequencing and analysis of the human genome. *Nature* **409**: 860–921.
17. Lebedev YB, Belonovitch OS, Zybrova NV, *et al.* 2000. Differences in HERV-K LTR insertions in orthologous loci of humans and great apes. *Gene* **247**: 265–277.
18. Lower R, Lower J, Kurth R. 1996. The viruses in all of us: characteristics and biological significance of human endogenous retrovirus sequences. *Proc Natl Acad Sci USA* **93**: 5177–5184.
19. Mamedov I, Batrak A, Buzdin A, *et al.* 2002. Genome-wide comparison of differences in the integration sites of interspersed repeats between closely related genomes. *Nucleic Acids Res* **30**: e71.
20. Medstrand P, Mager DL. 1998. Human-specific integrations of the HERV-K endogenous retrovirus family. *J Virol* **72**: 9782–9787.
21. Medstrand P, Landry JR, Mager DL. 2001. Long terminal repeats are used as alternative promoters for the endothelin B receptor and apolipoprotein C-I genes in humans. *J Biol Chem* **276**: 1896–1903.
22. Mi S, Lee X, Li X, *et al.* 2000. Syncytin is a captive retroviral envelope protein involved in human placental morphogenesis. *Nature* **403**: 785–789.
23. Schon U, Seifarth W, Baust C, *et al.* 2001. Cell type-specific expression and promoter activity of human endogenous retroviral long terminal repeats. *Virology* **279**: 280–291.
24. Sverdlov ED. 1998. Perpetually mobile footprints of ancient infections in human genome. *FEBS Lett* **428**: 1–6.
25. Sverdlov ED. 2000. Retroviruses and primate evolution. *Bioessays* **22**: 161–171.
26. Taruscio D, Mantovani A. 1998. Human endogenous retroviral sequences: possible roles in reproductive physiopathology. *Biol Reprod* **59**: 713–724.
27. Tristem M. 2000. Identification and characterization of novel human endogenous retrovirus families by phylogenetic screening of the human genome mapping project database. *J Virol* **74**: 3715–3730.
28. Turner G, Barbulescu M, Su M, *et al.* 2001. Insertional polymorphisms of full-length endogenous retroviruses in humans. *Curr Biol* **11**: 1531–1535.
29. Yi JM, Kim HM, Kim HS. 2001. Molecular cloning and phylogenetic analysis of the human endogenous retrovirus HERV-K long terminal repeat elements in various cancer cells. *Mol Cells* **12**: 137–141.
30. Yunis JJ, Prakash O. 1982. The origin of man: a chromosomal pictorial legacy. *Science* **215**: 1525–1530.

Copyright © 2002 John Wiley & Sons, Ltd. 
